# Lipid peroxidation and stress-induced signalling molecules in systemic resistance mediated by azelaic acid/AZELAIC ACID INDUCED1: signal initiation and propagation

**DOI:** 10.1007/s12298-024-01420-1

**Published:** 2024-03-15

**Authors:** Y. N. Priya Reddy, Ralf Oelmüller

**Affiliations:** 1 Matthias Schleiden Institute, Plant Physiology, Friedrich-Schiller University Jena, Dornburger Str. 159, D-07743 Jena, Germany; 2 Present Address: Max-Planck-Institute for Chemical Ecology, Hans-Knöll-Straße 8, D-07745 Jena, Germany

**Keywords:** Azelaic acid, AZELAIC ACID INDUCED1, Lipid peroxidation, Singlet oxygen, Lipoxygenase 2, Radicals

## Abstract

Systemic acquired resistance protects plants against a broad spectrum of secondary infections by pathogens. A crucial compound involved in the systemic spread of the threat information after primary pathogen infection is the C9 oxylipin azelaic acid (AZA), a breakdown product of unsaturated C18 fatty acids. AZA is generated during lipid peroxidation in the plastids and accumulates in response to various abiotic and biotic stresses. AZA stimulates the expression of *AZELAIC ACID INDUCED1* (*AZI1*), and a pool of AZI1 accumulates in the plastid envelope in association with AZA. AZA and AZI1 utilize the symplastic pathway to travel through the plasmodesmata to neighbouring cells to induce systemic stress resistance responses in distal tissues. Here, we describe the synthesis, travel and function of AZA and AZI1 and discuss open questions of signal initiation and propagation.

## Metabolites and proteins involved in systemic resistance

Local infections by pathogens cause resistance in distal tissues and protects them against subsequent pathogen attacks. The resistance in the whole plant is known as systemic acquired resistance (SAR) (Vlot et al. 2021). The resistance in the distal tissue requires mobile signal molecules which are generated at the local infection site and transported to uninfected distal tissues either through the plant body itself or via the air as volatiles (Kachroo and Kachroo 2020; Shine et al. 2019). Several signaling molecules which are at least partially transported through apoplastic or symplastic compartments or the phloem have been identified: this includes the lipid-derived oxylipin azelaic acid (AzA), glycerol-3-phosphate, pipecolic acid, N-hydroxy-pipecolic acid, dehydroabietinal, nitric oxide and reactive oxygen species (ROS) (Kim and Lim 2023; Gao et al. 2021, 2014; Siebers et al. 2016; Hartmann et al. 2017, 2018; Shine et al. 2019; Dempsey and Klessig 2012). The volatile monoterpenes α- and β-pinene and methyl salicylate is spread through the air to distal parts of the same plant but can also be perceived by neighbouring plants (Riedlmeier et al. 2017; Gong et al. 2023). Transport and function of these traveling signaling molecules are imbedded into a complex network with multiple interactions which included the proteins DEFECTIVE IN INDUCED RESISTANCE 1 (DIR1) and AZELAIC ACID INDUCED 1 (AZI1) (Yu et al. 2013). Here, we focus on the origin, transport and signalling of AZA and its interaction with AZI1. The involvement of AZA in whole plant immunity or signaling has been mainly studied in Arabidopsis infected with *Pseudomonas syringae* pv *tomato* (*Pst*) (Miranda de la Torre 2023; Banday et al. 2022; Witteck et al. 2014; Wang et al. 2014; El-Shetehy et al. 2015; Lim et al. 2016; Cecchini et al. 2019, Jung et al. 2009; Yu et al. 2013; Zoeller et al. 2012). AZA accumulation lowers the infection and disease spread also in systemic tissue of tomato, soybean (Korenblum et al. 2020) and crops (Saikia et al. 2020). The requirement of AZI1 for AZA function has been often demonstrated with *azi1* knock-out lines: all studies demonstrate that endogenous AZA or exogenously applied AZA requires AZI1 for systemic resistance (Gao et al. 2021; Dutton et al. 2019; Shine et al. 2019; Wang et al. 2016; Cecchini et al. 2015, 2019; Yu et al. 2013; Jung et al. 2009).

## AZA and its generation in plastids

AZA is a saturated linear C9 dicarboxylic acid (HOOC(CH_2_)_7_COOH) with multiple and diverse functions in eukaryotic organisms. In plants, AZA is mainly found in the plastid and plastid envelope (Pitzschke et al. 2014a), where it accumulates as a marker for lipid peroxidation under biotic and abiotic stress conditions (Adám et al. 2022). The plastid membranes contain lipid galactosides such as oleic acid, linolenic acid, or linoleic acid. These 18 carbon fatty acids contain a double bond at C9 which is hydrolysed to generate an AZA molecule (Zoeller et al. 2012; Yu et al. 2013; Wong et al. 2006; Gao et al. 2014). Zoeller et al. (2012) showed that three oxidation mechanisms are involved in AZA formation.

Singlet oxygen (^1^O_2_) is the major cause for lipid peroxidation and thus AZA formation under unstressed conditions dominates in green tissue (Triantaphylides et al. 2008; Triantaphylides and Havaux 2009; Farmer and Mueller 2013). In plastids, the major source of the nonradical electrophilic ^1^O_2_ is generated as a by-product during light capture at photosystem II, when energy is transferred from an excited chlorophyll molecule to ground-state O_2_ (Halliwell 2006; Kim and Apel 2013; Pospíšil 2016). ^1^O_2_ and other reactive oxygen species such as hydrogen peroxide and hydroxyl radical also arise during the interaction of light with chlorophyll precursors, i.e., protochlorophyllide or protoporphyrin IX, in the presence of molecular oxygen (Tripathy and Oelmüller 2012; Ryter and Tyrrell 1998; Fujii 2023). Photosynthetic organisms have developped quenching mechanisms to restrict ^1^O_2_ accumulation, e.g., via carotenoids, which convert ^1^O_2_ to ground-state O_2_, but under high light conditions, some ^1^O_2_ accumulates in or in the vicinity of the thylakoid membranes. In a non-catalytic reaction, one ^1^O_2_ generates one lipid hydroperoxide in the thylakoid membrane (Farmer and Mueller 2013).

Enzymatic lipid peroxidation is initiated by lipoxygenases (LOXs). LOX oxidize free fatty acids in the cytosol or chloroplasts, catalyze the hydroperoxidation of C-18 unsaturated fatty acids, thereby initiating several oxylipin pathways including the jasmonate and hydroperoxide lyase pathway (Mosblech et al. 2009) which results in the synthesis of intermediates for several defense-related products, including AZA and the jasmonic acid (Vick and Zimmermann 1987; Rosahl et al. 2005). In case of linolenic acid, the enzyme catalyzes the stereospecific oxygenation of the position 13 of linolenic acid to form linolenic acid 13-hydroperoxide. Relevant for AZA biosynthesis is the plastid localized LOX2, which participates in lipid peroxidation, but knock-out lines demonstrate that this enzyme is not essential for AZA biosynthesis, at least after pathogen attack (Zöller et al. 2012). *LOX* expression is developmentally controlled and stimulated in response to wounding, pathogen attack and water deficit (Bell and Mullet 1991, 1993; Siedow 1991; Koch et al. 1992; Ohta et al. 1991). The enzyme is involved in many biotic stress responses including priming processes (Losvik et al. 2017; Rustgi et al. 2019; Zhao et al. 2023), as well as drought, cold and salt stress (cf. De Domenico et al. 2012; Nieto-Garibay et al. 2022; Liu et al. 2017; Du et al. 2013; Shi et al. 2022). Finally, hydroperoxide lyases catalyse the cleavage of C–C bonds in the hydroperoxides to generate oxylipins, including AZA (Matsui et al. 2006).

Radical-catalyzed lipid peroxidation is mainly initiated by the the radical source H_2_O_2_. In the presence of irons, H_2_O_2_ is degraded to hydroxyl radicals (HO^•^) and superoxide anion radicals (OO^•−^) (Halliwell 2006; Mittler et al. 2004). HO^•^, but not OO^•−^, abstracts hydrogen from fatty acids, which generates various lipid peroxides in the presece of oxygen. The free radicals break down 18:1, 18:2 and 18:3 fatty acids. These processes can also be catalyzed by other radicals, such as reactive nitrogen species. Peroxynitrite (^•^ONOO), the reaction product of nitric oxide (NO) and OO^•−^ (Vandelle and Delledome 2011) reacts with CO_2_ to generate carbonate radicals (Radi 1998). NO triggers synthesis of various ROS species (superoxides, hydroxyls, ^1^O_2_ or H_2_O_2_) and all radicals act additively to catalyze the oxidation of free C18 unsaturated fatty acids to generate AZA (Wendehenne et al. 2014; cf. also Yu et al. 2013). Different ROS species are also generated during abiotic stress (Li and Kim 2021; and ref. therein) indicating that both biotic and abiotic stress may contribute to AZA production via free radicals.

The pathways do not operate completely independent of each other. For instance, during pathogenesis, photosystem II activity is normally inhibited with results in an increased accumulatioin of ^1^O_2_ (Triantaphylides et al. 2008; Triantaphylides and Havaux 2009). Furthermore, ^1^O_2_-mediated lipid fragmentations generate radicals, which cause additional membrane and lipid damage. Zoeller et al. (2012) demonstrated that the free radical-catalyzed galactolipid fragmentation mechanism is mainly responsible for AZA formation in Arabidopsis after pathogen (*Pseudomonas syringae* pv tomato DC3000) infection. Therefore, besides functioning a mobile defense signals for whole plant immunity, AZA is a marker for lipid oxidation (cf. Cecchini et al. 2019; Shine et al. 2019; Gao et al. 2021).

## AZI1 and AZA transport

The transport of AZA or the AZA signal requires the lipid transfer protein AZI1, a member of the hybrid proline-rich protein (HyPRP) family. A pool of AZI1/EARLY ARABIDOPIS ALUMINIUM INDUCED1 (EARLI1), a close paralog of AZI1, localizes to the plastid envelope. Mainly based on experiments where AZA was exogenously applied to leaf tissue, it was shown that AZA induces *AZI1* and *EARLI1* expression in the nucleus (Jung et al. 2009).

**Fig. 1:**
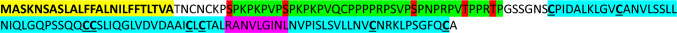
AZI1 consists of secretion signal (yellow), a proline-rich domain (PRD, green), and a characteristic eight-cysteine-motif (8-CM) segment (blue) (cf. Jose-Estanyol et al. 2004). The secretion signal and the PRD also function as a bipartite non-cleavable N-terminal transit sequence for plastid import that harbors a transmembrane domain and anchors the protein to membranes. The cysteines in the 8-CM segment are underlined and bold. Five putative MAPK phosphorylation sites (serine, S; threonine, T) are in red (Pitzschke et al. 2016). The putative MAPK interaction sites with the consensus (R/K x_2–6_ L/IxL/I) is in purple (Cecchini et al. 2021). The hydrophobic C-terminal 8-CM domain is also present in lipid transfer proteins, amylase inhibitors and 2S albumins (Dvorakova et al. 2007; Jose-Estanyol et al. 2004)

AZI1 contains an amino terminal hydrophobic domain (a signal peptide (cf. below)) which is followed by the proline-rich region. These two domains (signal peptide and proline-rich region) can be considered as a non-cleavable bipartite N-terminal signature that shares features with plastid transit peptides, and the transmembrane domain of the signal peptide which anchors the protein to membranes (Fig. 1). The transmembrane domain is required for the ring‐like pattern of plastid membrane association, i.e., AZI1´s association with the plastid envelope. Cecchini et al. (2021) showed that the signal peptide/hydrophobic domain and the proline-rich region are required for targeting of AZI1 to the plastid outer envelope membrane. AZI1´s paralog EARLI show the same protein structure and targeting features. Targeting of AZI1 and EARLI1 to chloroplasts is increased during SAR (Cecchini et al. 2021). Application of flg22 (a 22 amino acids-long peptide from the bacterial flagellin that functions as pathogen associated molecular pattern) results in elevated AZI1/EARLI1 protein levels and promotes their protein pools in the plastid fraction. Also the defense-associated MITOGEN-ASSOCIATED PROTEIN KINASE3 (MAPK3) and -6 are involved in promoting the accumulation of AZI1 at plastids (Cecchini et al. 2021). MAPK3 and -6 can phosphorylate AZI1 in vitro and in vivo (Pitzschke et al. 2014a; Cecchini et al. 2021) (Fig. 1), which promotes entry of AZI1 into and sorting at the organelle, in particular under stress (cf. below) (Cecchini et al. 2021). Stress induces AZA production and AZI1 could facilitate the movement of AZA, and potentially also other plastid oxylipins related to stress resistance from the plastid outer membrane system to the endoplasmatic reticulum (Cecchini et al. 2021). The plastid outer membrane functions as a defense platform against several biotic and abiotic stresses, since it contains enzymes for the synthesis of fatty acids and for a variety of fatty acid derivatives (Breuers et al. 2011). Digalactosyldiacylglycerol synthase for fatty acid biosynthesis (Froehlich et al. 2001a) and enzymes for the breakdown of fatty acid hydroperoxides are located in the chloroplast envelope membranes (Blée and Joyard 1996; Froehlich et al. 2001b). For instance, the hydroperoxide lyase is an outer envelope membrane enzyme that catalyzes the first step towards defense-related aldehydes (Blée and Joyard 1996; Howe and Schilmiller 2002; Kishimoto et al. 2008). AZA might be generated at the envelope membrane or transported to it from the thylakoid membrane. The radical precursor H_2_O_2_, which is the major source for AZA formation after pathogen attack (Zoeller et al. 2012), is generated by the NADH oxidase at the apoplastic side of the plasma membrane after pathogen attack and might have better or faster access to the outer envelope membrane than to the thylakoid membranes, because diffusion of H_2_O_2_ across membranes (plasma membrane alone or plasma membrane and inner envelope membrane of chloroplasts) is limited (Bienert et al. 2006). Transport of AZA from the thylakoid membrane to the envelope membrane requires passage through the inner envelope membrane, a process that most likely involves a transport molecule. The dynamic non-covalent interaction between the plastid outer membrane and the endomembrane system play important roles in lipid trafficking and trafficking of membrane-bound signalling molecules (Breuers et al. 2011). This allows AZA movement within the cell membranes and translocation to neighbouring cell via plasmodesmata (cf. below). How AZI1 supports AZA trafficking is not clear.

Gel retardation assays showed that AZI1 is posttranslationally modified. AZI1 forms protein complexes and MAPK3 (Pitzschke et al. 2014b) and the potential phosphorylation sites in AZI1 are located in the proline-rich region (Fig. 1). Pitzschke et al (2014b) demonstrated that phosphorylation is physiologically relevant. The *azi1* mutant is hypersensitive to salt stress, while *AZI1*-overexpressor lines are more tolerant than the wild-type. Since *AZI1* overexpression in the *mapk3* background partially alleviates the salt-hypersensitive phenotype MAPK3 which further involved in the AZI1-conferred robustness against this stress. Furthermore, the proline-rich region found in HyPRPs shows similarities to arabinogalactan proteins which are modified by proline hydroxylation and subsequent O-glycosylation. This posttranslational modification occurs also in AZI1 since inhibition of prolyl hydroxylase reduced the apparent protein size of AZI1 (Pitzschke et al. 2016). These protein modifications are stress-independent and unrelated to the phosphorylation by MAPKs. It remains to be determined whether this modification affects other physiological processes, or AZI1 sorting in the cell.

Zöller et al. (2012) showed that local AZA production was not compromised in the *azi1* mutant, suggesting that AZA accumulation is independent of AZI1. Other family members have not yet been tested. Likewise, whether AZI1 accumulation at the plastids is affected by AZA, has also not yet been investigated. This also holds true for the AZI1´s paralog EARLI1. Like AZI1, EARLI1 accumulates at the plastid envelope during defense, and the regulation of the expression of the *EARLI1* gene exhibits similarities to that of *AZI1*. Furthermore, EARLI1 is also involved in systemic defense priming and SAR (Cecchini et al. 2021).

AZI1 is a member of the HyPRP superfamily, with 28 members in Arabidopsis. All HyPRPs have a transmembrane domain, a proline-rich region, and a lipid transfer protein domain (a characteristic eight-cysteine-motif segment; cf. Jose-Estanyol et al. 2004) (Fig. 1). The precise subcellular location(s) and function(s) for most HyPRP family members are unknown (Banday et al. 2022). Besides AZI1, also HyPRP members have a pool of proteins that target plastid outer envelope membranes via their proline-rich domains. Two HyPRPs are associated with thylakoid membranes (Banday et al. 2022), and AZI-LIKE2 (AZL)2, AZL13, AZL14 and ELP (EXTENSIN-LIKE PROTEIN) are outer envelope membrane proteins. AZL3 and DRN1 (DISEASE RELATED NONSPECIFIC LIPID TRANSFER PROTEIN1) are either thylakoid or/and envelope membrane proteins. Most of the plastid- and nonplastid-localized family members also have pools that are localized to the endoplasmic reticulum, plasma membrane, or plasmodesmata (Banday et al. 2022). Some of the HyPRPs are cell-wall structural proteins, and they are either positive or negative regulators of abiotic and biotic stress responses in different plant species (Saikia et al. 2020). In crop plants, they participate in cold, drought, salt and oxidative stress responses, down-regulate ROS scavenging genes or participate in basal defense against pathogens (cf. Table 1 in Saikia et al. 2020). Besides its plastid/envelope membrane localisation, also part of AZI1 is found in the apoplast (Pitzschke et al. 2016). The N-terminal secretion signal directs an AZI1-fluorescent protein fusions to the endoplasmatic reticulum (Yu et al. 2013) and the fusion protein is further secreted via the secretory pathway to the cell surface (Pitzschke et al. 2014b; Zhang and Schlappi 2007). Furthermore, proteomic studies identified AZI1 in plasmodesmata and the plasma membrane (Fernandez-Calvino et al. 2011; Mitra et al. 2007, 2009). Pitzschke et al. (2016) also showed that a proportion of AZI1 is secreted by protoplasts, however, the majority of the protein remained in the protoplast fraction. This demonstrates similarities between AZI1 and apoplastic HyPRPs with stress-regulatory functions, and suggests that translocation of AZI1 or an AZI1-derived mobile signal in the establishment of SAR could also occur via the apoplastic space, although to a lesser extent than the plastid-originated signaling pathway. Two systemic signaling pathways, one starting in or at plastids and another one from secreted AZI1, exists, has to be investigated. Moreover, the role of AZA in a scenario with secreted AZI1 is not clear; it is conceivable that AZA only induces *AZI1* expression to promote the apoplastic AZI1 pool. Furthermore, the unique function of AZI1 among the HyPRPs is not well understood. Although knock-out mutants showed that AZI1 cannot be replaced by other HyPRPs including EARL1, it has not yet been investigated in details whether (or to what extent) other HyPRPs (except EARLI1, cf. Cecchini et al. 2021) can induce systemic signalling.

## AZA (signal) movement to systemic tissue and priming

Threat information travels from the local site exposed to a threat stimulus to distal tissues, which somehow stores the threat information, a process called priming. In primed tissues, the defense responses are not activated yet. However, if primed tissue is exposed to the same or similar threat, that induced the primed state, the response differs from that of the unprimed tissue, since it activates induced systemic resistance or SAR programs (Conrath et al. 2015; Fu and Dong 2013; Oelmüller 2021). Defense priming mediated the AZA/AZI1 results in a faster and stronger activation of defense and antioxidant genes, genes for phytohormones and enzymes involved in the synthesis of defense-related metabolites including volatiles. In tobacco cells, genes for pathogenesis-related proteins and enzymes involved in phenylpropanoid pathway and chlorogenic acid metabolism as well as signal transduction components responded to AZA application in systemic tissue (Djami-Tchatchou et al. 2017). Defense related metabolites included caffeoylputrescine glucoside and related secondary compounds. Salicylic acid is the major phytohormone activated upon AZA application. This raises the question how AZA induces a primed state. Since AZA or the AZA-derived signal does not directly activate defense genes, they should target molecules which alter the physiological state in the primed cell. A possible scenario has been recently described by Miranda de la Torre et al. (2023). Priming involves chromatin modifications for a faster/stronger activation of defense genes (Miranda de la Torre et al. 2023). The chromatin regulator MORPHEUS MOLECULE1 (MOM1) functions as a priming factor which affects the expression of several immune receptor genes. AZA treatments reduce *MOM1* expression in systemic tissues and lower MOM1 levels sensitize the primed tissue to biotic stresses. In plants exposed to stressful conditions, the decrease in MOM1 facilitates the upregulation of immune receptors, which improves the perception of future attacking pathogens and the amplification of the plant defense responses. Therefore, MOM1 is as a chromatin factor that negatively regulates the defense priming induced by AZA.

Besides its involvement in defense priming, AZI1 is also required for the reduction of stomata density to restrict Pseudomonas entry, as shown by Dutton et al. (2019). This suggests that–besides relatively fast immune priming in distant tissue—AZA also participates in long-term developmental programs.

Translocation of the AZA-dependent information from local to distal tissue can occur in three ways. The compounds either travel directly from the local application site to the distal tissue, or activates traveling of other signalling compounds, or induces signalling events that activate its own de novo synthesis along the traveling path and ultimately in the distal tissue (Hartmann et al. 2018; Wang et al. 2018; Cecchini et al. 2019; Vlot et al. 2021). Since AZA is generated by lipid peroxidation, de novo synthesis along the traveling path or in distal tissue requires cells where lipid oxidation occurs. Jung et al. (2009) were among the first who proposed that AZA is transported to non-infected systemic leaves after *P. syringae* infection. However, Zoeller et al. (2012) showed that the AZA level in the systemic leaves 24 h after local infection was not elevated in comparison to the levels found in non-infected control leaves. Furthermore, AZA-inducible *AZI1* expression was not stimulated in the systemic leaves.

AZA is found in roots and leaves, and Cecchini et al. (2015) showed that exogenously applied ^14^C-AZA can move within the plant body. Movement of label from one leaf (the application site) to total systemic tissues (aerial stem/leaves and roots) was significantly reduced in *azi1* and *earli1-1* compared to wild-type plants (Cecchini et al. 2015). In wild-type, a large amount of the signal that moved within aerial tissues was detected in very young leaves. Interestingly, a lot of the label also moved systemically from leaves to the roots. The *azi1* and *earli1* mutants showed significant decreases in label uptake into leaf discs (∼25%) compared to the wild-type, when AZA was applied exogenously. The authors concluded that movement and uptake of AZA (and possibly AZA derivatives) partially depends on AZI1 and EARLI1. However, Cecchini et al. (2019) showed that deuterium-labeled AZA applied to the roots does not move to aerial tissues, although AZA application to roots triggers systemic immunity in leaves. This suggests that AZA can travel root-, but not shootwards. The authors postulated an AZI1/EARLI1/MAPK3/-6-dependent pathway and the AZA effects may involve additional mobile signals. Apparently, translocation of exogenously applied AZA depends on the directions and tissue. Since AZA-induced immune responses in distal tissues are not always associated with its translocation to this tissue or with elevated AZA levels in this tissue, the involvement of additional molecules is likely. Furthermore, whether AZA travels alone or in association with AZI1, remains to be investigated.

Direct or indirect interactions of AZA with other systemic signaling molecules involved in SAR responses have been reported which might be involved in the translocation of the information to distal tissue. Besides EARLI1 and the above mentioned pipecolic acid, N-hydroxy-pipecolic acid, dehydroabietinal, glycerol-3-phosphate, the monoterpenes α- and β-pinene, methyl salicylate, NAD(P) and DIR1, the hormone salicylic acid, the free radicals NO and ROS have been described (Gao et al. 2021; Huang et al. 2023; Riedlmeier et al. 2017; Shine et al. 2019; Yu et al. 2013; Dempsey and Klessig 2012; El-Shetehy et al. 2015; Gao et al. 2015). Salicylic acid acts in parallel with the two radical signals NO and ROS, and simultaneous activation of the salicylic acid and NO/ROS pathway is essential for full SAR responses. NO/ROS acts upstream of AZA, as well as glycerol-3-phosphate (Wang et al. 2014). Yu et al. (2013) demonstrated that a feedback regulatory loop between glycerol-3-phosphate and the lipid transfer protein DIR1 and AZI1 mediates AZA-induced systemic immunity. Wang et al. (2016) showed that soluble carbohydrates might function as signal substances in the systemic immunity of Arabidopsis. The expression of the sugar signaling genes (*SUS1, -2*, *-3*, *-6*, *SUT1*, *HXK1*, *-2*, *SNRK1.1*, -*1.2*, -*1.3*, *ERD6*, *TPS1*, *TOR* and *bZIP11*) in local and distal leaves after infection of avirulent *P. syringae* was changed in plants with modulated AZI1 activities (knock out and overexpressor lines), indicating that sugar-related genes are involved in regulation of the systemic immunity mediated by AZI1. This suggests an extended cross-talk between systemic signalling molecules and the primary sugar metabolism, raising the question how AZI1 is integrated into the network.

Besides AZA´s direct participation in defense priming, HyPRPs are also involved in balancing beneficial and pathogenic traits in symbiotic interactions. For instance, HyPRPs regulate the interaction with the plant growth-promoting rhizobacteria *Pseudomonas simiae* WCS417 in the roots to influence colonization, root system architecture, and/or biomass. Therefore, HyPRPs have broad and distinct roles in immunity, development, and growth responses to microbes and reside at sites that may facilitate signal molecule transport (Banday et al. 2022). Furthermore, some mutants of this family are also affected in both induced systemic resistance and SAR, suggesting overlapping functions with AZI1/EARLI1.

## Symplastic transport of AZI1 to the phloem

Movement of small proteins or metabolites to systemic tissues occurs often via the phloem (Dinant and Lemoine 2010). Uploading of AZI1 to the phloem occurs via the symplastic transport (Lim et al. 2016) and the protein reaches the phloem via sorting signals which direct it from the outer plastid membrane to the endoplasmic reticulum and plasmodesmata which transverse the cell wall and join the adjacent cells. Being a lipid-binding and membrane-bound protein, AZI1 appears to travel to the phloem companion cells via direct membrane–membrane contact sites, which have been identified at the outer plastid membrane, the endoplasmatic reticulum and the plasma membrane. In case of lipid transfer proteins such as AZI1 these contact sites also allow exchange of the bound lipids (Breuers et al. 2011; Wang and Benning 2012; Helle et al. 2013, Cecchini et al. 2015). Lim et al. (2016) demonstrated that two plasmodesmata-localized proteins regulated SAR function in both, signalling and plasmodesmata gating of AZI1. While PLASMODESMATA LOCALIZING PROTEIN1 (PDLP1) interacts with AZI1, is required for endoplasmatic reticulum-specific localization of AZI1, and contributes to the intracellular portioning of the protein, PDLP5, which impairs plasmodesmata permeability and thus transport of AZA to the neighboring cell. PDLP1 interacts with PDLP5 which regulates the symplastic transport and plasmodesmata gating (Lee et al. 2011; Lim et al. 2016). *PDLP5* knockout mutants increase and overexpressor lines restrict general plasmodesmata permeability (Lee et al. 2011; Wang et al. 2013). Importantly, the *pdlp1* mutants contained reduced AZI1-GFP protein levels, although the *azi1*-*gfp* transcript levels were not affected (Lim et al. 2016). This suggest that PDLP1 affect the stability of AZI1. Furthermore, in the *pdlp1* mutant, AZI1 was primarily localized to the outer plastid membrane, whereas in wild-type plants, the majority of the protein is located in extraplastidic compartments. The studies by Lim et al. (2016) highlight the importance of PDLP1 for AZI1 stability and its traveling from the plastids to the plasmodesmata.

Jung et al. (2009) showed that AZI1 is important for generating vascular sap that confers disease resistance. AZA and petioles exudates failed to induce systemic immunity in *azi1* plants. Pathogen-induced exudates from *azi1* were inactive when applied to wild-type plants. Therefore, AZI1 modulates production and/or translocation of a mobile signal(s) during SAR. The AZI1 target in the vascular sap is unknown so far.

## *AZI1* gene activation and abiotic stress

*AZI1* expression is stimulated by exogenously applied AZA (Jung et al. 2009). Whether stimulation of the endogenous AZA levels due to lipid peroxidation under stress *in planta* is a prerequisite for the *AZI1* expression is not known.

The best studied biological stimulus for *AZI1* activation comes from Arabidopsis leaf infiltration assays with *Pseudomonas syringae* (cf. Arabidopsis eFP Browser). Similarly effective is the bacterial effector flg22. Late stimulation of *AZI1* expression was also observed after co-cultivation of Arabidopsis seedlings with *Hyaloperonospora arabidopidis* (cf. Arabidopsis eFP Browser). Relatively little is known about the role of AZI1 for other pathogenic or beneficial plant–microbe interactions, including pathogenic fungi, nematodes, insects, mycorrhizal fungi and beneficial endophytes, although some of them can produce AZA. For instance, AZA is produced by *P. syringae* (Javvadi et al. 2018) or the root colonizing endophytic fungus *Piriformospora indica* (Kundu et al. 2022), however, whether microbe-synthesized AZA activates *AZI1* in plants or participates in defense priming in plants, is not known.

Besides biotic stress, expression profiles demonstrate that the *AZI11* mRNA level responds also to abiotic stress, however functional analyses are often missing. Xu et al. (2011) showed that the *AZI1* transcript level, as well as that of its paralog *EARLI1* (Zhang and Schäppi 2007), increases after exposure of Arabidopsis seedlings to cold. The increase of the *AZI1* mRNA level was slow, since more than 6 h at 4 °C was required for the induction. The mRNA level declined to basal levels when the plants were transferred back to room temperatures. Overexpression of *AZI1* resulted in reduced electrolyte leakage during freezing damage, while *AZI1* knockdown and knockout lines showed increased tendencies in cellular damage after freezing treatment. When *Saccharomyces cerevisiae* cells were transformed with *AZI1* under the control of *GAL1* promoter, the survival rate of yeast cells harbouring AZI1 increased after freezing treatment. This demonstrates that AZI1 might be multifunctional and associated with cold tolerance of Arabidopsis (Xu et al. 2011). The involvement of AZI1 in cold stress adaptation is further supported by expression profiling of mutants manipulated in cold stress-acclimation genes. Similar results were obtained for *Thelunsiella salsuginea* (Wong et al. 2006).

The ICE-CBF-COR (Inducer of CBF Expression—C-repeat Binding Factor—Cold Regulated) signalling pathway is an important regulator for cold-stress acclimation (Gusain et al. 2023). CBF overexpressors show increased cold tolerance and high levels of *AZI1* gene expression (Wong et al. 2006). Likewise, *DEHYDRATION-RESPONSIVE-ELEMENT-BINDING PROTEIN1* (*DREB1*) genes are induced by cold stress, and overexpression of *DREB1* induced strong expression of other stress-responsive genes, resulting in increased tolerance to high-salt and freezing stresses (Ito et al. 2006). Among the genes which are up-regulated in the *DREB1* overexpressor lines after exposure to cold stress is *AZI1* (Maruyama et al. 2004).

Besides cold, *AZI1* is involved in salinity stress tolerance. Pitzschke et al. (2014b) showed that *azi1* mutants are hypersensitive to salt. At 150 mM salt stress, only 7% of the *azi1* mutant seeds, 70% of wild-type seeds, and 90% of the seeds of *AZI1* overexpressor lines germinated. Furthermore, *AZI1* overexpressors thrived better than the *azi1* mutants under high salt conditions. Another example provides mutants in with the salt stress signalling gene *ZINC FINGER OF ARABIDOPSIS THALIANA12* (*ZAT12*) was manipulated. The overexpressor lines performed better under salt stress and this was associated with the higher expression levels of *AZI1* and *EARLI1* (Davletova et al. 2005). Furthermore, exposure of the salt tolerant xero-halophyte *Haloxylon salicornicum* to salt stress resulted lower stearic acid and palmitic acid levels. Panda et al. (2021) speculated that breakage of lipid membranes might lead to higher accumulation of AZA. In conclusion, AZA is also involved in abiotic stress tolerance in various plant species, such as cold (Davletova et al. 2005) and salt tolerance (Atkinson et al. 2013). When Arabidopsis seedlings are exposed to simultaneous biotic and abiotic stresses, *AZI1* was down-regulated in leaves and conferred drought susceptibility when overexpressed (Atkinson et al. 2013). More functional analyses are required to understand the role of AZI1 in abiotic stress responses.

## Conclusion and open questions

The AZA/AZI1 pathway is involved in both biotic and abiotic stress responses in plants, and a comparative analysis of both stimuli might be helpful to throw more light on the molecular mechanism of systemic resistance. AZA accumulates in response to lipid peroxidation and Zöller et al. (2012) showed that lipid peroxidation is predominantly confined to plastid lipids comprising galactolipid and triacylglyceride species during the interaction of Arabidopsis with *P. syringae*, i.e. biotic stress. ^1^O_2_ was identified as the major cause of lipid oxidation under basal conditions, while LOX2- and free radical-catalyzed lipid oxidation substantially contribute to the increase upon pathogen infection (Zöller et al. 2012). It remains to be determined, whether all AZI1-mediated biotic and abiotic stress responses are linked to AZA and lipid peroxidation in the plastids. Barely anything is known about the role of AZA in AZI1-dependent abiotic stress responses and whether these responses are restricted to local tissues or operate systemically. Systemic signal propagation induced by abiotic stresses might be agriculturally important, e.g. for crop plants with roots in cold soil and aerial parts exposed to extreme heat. Finally, the role of the secreted AZI1 in the apoplast or at the plasma membrane for systemic immune responses and local abiotic stress responses has not yet been studied. This is particularly interesting since other members of the HyPRP family which are found in the apoplast, participate in abiotic stress responses (Saikia et al. 2020).

The initiation of the AZA/AZI1 signaling at plastids needs to be investigated in more details. AZA is present in roots and shoots, but the plastids and the intraorganellar membrane structure as the site of lipid peroxidation differ substantially in two types of plastids. In both organs, AZI1 has been shown to be associated at least in part with plastids. In particular, in the aerial tissue, HyPRPs are mainly found in epidermal cells. Their plastids play key roles in defense against microbes (cf. Banday et al. 2022). Investigating the role of root plastids for the generation of AZA and the AZA/AZI1 interaction is important for unravelling the function of the signalling compounds in roots. Furthermore, *AZI1* and *EARLI1* expression is strongly down-regulated in roots upon colonisation by fungi (Banday et al. 2022), whereas this was not observed for other *HyPRP* genes. This raises the question whether AZI1/EARLI1 might have also other functions in roots, e.g., by controlling root colonisation or entry of fungal hyphae into the roots. Banday et al. (2022) have already demonstrated that HyPRPs regulate the interaction with the plant growth-promoting rhizobacteria *Pseudomonas simiae* WCS417 in the roots to influence colonization, root system architecture, and/or biomass. Further studies are required to understand the differences in the regulation of these *HyPRP* genes, as well as function and signalling of the proteins in roots and shoots upon pathogenic and beneficial microbial attacks.

Disruption of galactolipids in the plastid membranes by lipid peroxidation generates breakdown products including the oxo-acid AZA which protrude to the aqueous phase. In particular, during membrane repair, this promotes AZA release from the membrane and mobility to neighbouring membranes, either alone or complexed by AZI1. The role of AZI1 for the movement of AZA between membranes requires further attention. Whether membrane disruption during the oxidative process plays a role for AZA movement, should be investigated.

**Fig. 2:**
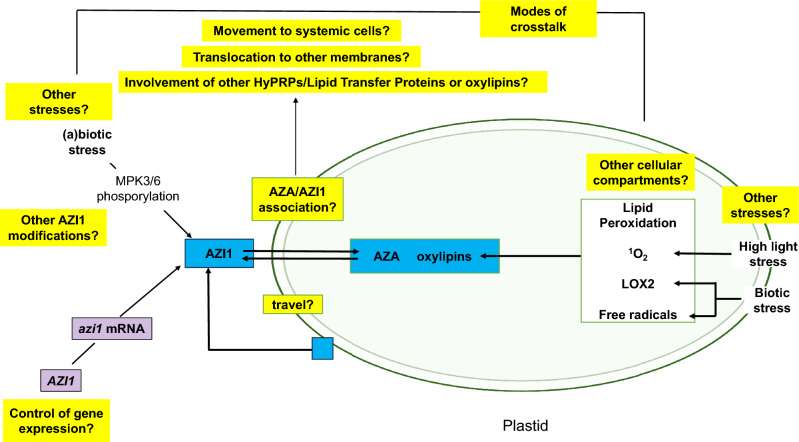
A model highlighting the open questions (yellow) during early events required for the generation of AZA and AZI1 in response to biotic and abiotic stress. For details, cf. text

Based on the current knowledge about the regulation of the pathway, localisation of AZI1 in or at plastids and its trafficking to other cellular membranes are early events that proceed the activation of the systemic movement of the priming signal of AZA/AZI1 to distal tissue (cf. Cecchini et al. 2021). Plastid targeting of AZI1 is promoted by MAPK3/6, which is activated by biotic (Pitzschke et al. 2009) and abiotic stresses (e.g. Li et al. 2014). Besides MAPK3/6 activation, biotic and abiotic stresses also generate ROS (Takata et al. 2020; Rodriguez et al. 2010), which–in turn—promote plastid-association of AZI1 via MAPK3/6 signaling, but also lipid peroxidation in plastids which generates AZA. It would be interesting to know how the AZI1 and AZA generation is coordinated (Fig. 2).

A main question centers around the long-distance transportation of AZA or the AZA signal to systemic tissue. If AZA can travel root-, but not shootwards, one has to postulate different mechanisms for the propagation of the information from the roots to the shoots and from the shoots to the roots. AZA is not water soluble and it is well known that membrane contact sites between plastid envelopes, endoplasmatic reticulum, plasma membrane and membrane material at the plasmodesmata are the sites of exchange of small molecules, including AZA (Andersson et al. 2007; Toulmay and Prinz 2011; Li et al. 2020). Small signaling molecules can be rapidly transported to the systemic tissues through the phloem (Gao et al. 2021), however, this is not yet clear for AZA. In addition, the role of AZI1 during the long-distance transport of AZA is not yet understood. If AZA cannot travel shootwards, which signalling compounds are activated to establish SAR in the aerial parts, how are they activated in the roots by AZA/AZI1 and how are they traveling? What is the role of salicylic acid in this scenario?

AZA is only one of the closely related oxylipins, which are generated during lipid peroxidation. Enzymatic oxidative fragmentation of 18:3 lipids results in the accumulation of 9-oxononanoic acid and nonadienal, besides AZA (Zöller et al. 2012; Matsui 2006). The biotin precursor pimelic acid is an important lipid peroxidation product which accumulates during free radical-catalyzed galactolipid fragmentation and its accumulation occurs independently of the LOX2 pathway (Zöller et al. 2012). However, pimelic acid did not induce SAR (Wittek et al. 2014). 9-Hydroperoxy octadecadienoic acid and 9-oxo nonanoic acid can be considered as precursors of AZA and oxidation of exogenously applied 9-oxo nonanoic acid to Arabidopsis establishes SAR, suggesting that it is oxidized to AZA (Wittek et al. 2014). Wittek et al. (2014) showed that—besides AZA—the C9 lipid peroxidation product 9-oxo nonanoic acid is linked to systemic rather than local resistance and the authors suggested that salicylic acid and its upstream regulator ENHANCED DISEASE SUSCEPTIBILITY1 (EDS1) directly or indirectly promotes the accumulation of 9-oxo nonanoic acid, AzA, or one or more of their common precursors possibly by activating one or more pathways that either result in the release of these compounds from galactolipids or promote lipid peroxidation. Furthermore, for several oxylipins, induction of defense responses and root growth inhibition has been reported (Vellosillo et al. 2007; Blée 2002; Prost et al. 2005). Therefore, the role of these lipid peroxidation products needs to be investigated in more details. So far, the AZA/AZI1couple is in the main focus in the field, while the role of EARLI1 and other lipid transfer proteins/HyPRPs in this scenario have been less studied (cf. Banday et al. 2022). As mentioned above, several members of the HyPRP family are involved in stress responses. Whether they can be activated by lipid peroxidation products is not yet known. It is possible that other lipid transfer proteins/HyPRPs have overlapping functions with AZI1, although they are located in different membranes or cellular compartments? It remains to be investigated whether not yet investigated oxylipin/HyPRP combinations may facilitate systemic resistance in response to specific biotic or abiotic stresses.
